# Gene therapy of adeno‐associated virus (AAV) vectors in preclinical models of ischemic stroke

**DOI:** 10.1111/cns.14392

**Published:** 2023-08-08

**Authors:** Jing Wang, Mengna Zhu, Jingyi Sun, Lina Feng, Mingfeng Yang, Baoliang Sun, Leilei Mao

**Affiliations:** ^1^ Medical College of Qingdao University Qingdao China; ^2^ Institute for Neurological Research, The Second Affiliated Hospital School of Basic Medical Sciences of Shandong First Medical University & Shandong Academy of Medical Sciences Taian China; ^3^ Department of Spinal Surgery Shandong Provincial Hospital Affiliated to Shandong First Medical University Jinan China

**Keywords:** adeno‐associated virus, gene therapy, ischemic stroke

## Abstract

Stroke has been associated with devastating clinical outcomes, with current treatment strategies proving largely ineffective. Therefore, there is a need to explore alternative treatment options for addressing post‐stroke functional deficits. Gene therapy utilizing adeno‐associated viruses (AAVs) as a critical gene vector delivering genes to the central nervous system (CNS) gene delivery has emerged as a promising approach for treating various CNS diseases. This review aims to provide an overview of the biological characteristics of AAV vectors and the therapeutic advancements observed in preclinical models of ischemic stroke. The study further investigates the potential of manipulating AAV vectors in preclinical applications, emphasizing the challenges and prospects in the selection of viral vectors, drug delivery strategies, immune reactions, and clinical translation.

## 1 INTRODUCTION

Stroke is a major health issue globally, being the second leading cause of death and the third leading cause of death and post‐stroke disability combined.^1^, ^2^ A significant proportion of new stroke cases are characterized as ischemic stroke, which can result in varying degrees of neurological deficits and complications, severely compromising patients' personal and social quality of life. Despite globally recognized pathology with the highest mortality and morbidity, the current treatment strategies for stroke are mainly ineffective. Although different treatment options have been studied in different randomized controlled trials, the only option that successively gained the interest of practitioners is treating ischemic stroke by intravenous thrombolysis or mechanical thrombectomy during the acute stroke attack to restore blood flow.^3^, ^4^ However, the narrow treatment window and strict criteria for selecting stroke patients are inherently associated demerits of that strategy,^5^, ^6^ which demands other novel treatment methods for improving stroke treatment outcomes.

Gene therapy has emerged as a novel and innovative approach for treating stroke, involving DNA or RNA as pharmacological agents for manipulating target genes or disease treatment using specific vectors,^7^, ^8^ particularly adeno‐associated viruses (AAVs). AAVs are small and dependent viruses that can transduce both dividing and non‐dividing cells, with aided merits of simplicity, operation ease, high safety and transduction efficiency, tissue or cell‐targeting specificity, and long‐lasting expression.^9^, ^10^ AAV‐mediated gene therapy applications have been approved for various genetic diseases and cancers, and currently, there are 14 ongoing or completed clinical studies focusing on central nervous system (CNS) diseases such as spinal muscular atrophy, Parkinson's disease, Sanfilippo syndrome, and Carnarwan disease.^11^, ^12^, ^13^ Although clinical trial reports of AAV therapy for ischemic stroke are scarce, the growing preclinical studies on AAV‐mediated stroke gene therapy and significant progress in genetic engineering are envisaged to provide hope for AAV vectors to be used for treating stroke patients.

This review delves into the biological attributes of AAV as gene vectors, followed by analyzing their potential as a carrier for gene therapy in preclinical animal models of ischemic stroke and evaluating their impact. Furthermore, the manipulation selection of AAV vectors in preclinical stroke applications, challenges, and prospects in selecting viral vectors for drug delivery, immune response progress, and clinical implementation are also presented.

## 2 AAV AS A GENE DELIVERY VECTOR

Viral vectors have received immense attention and played a pivotal role in gene therapy in the previous two decades.^14^ Reverse transcriptase viral vectors can mediate the generation of human neural stem cell (NSC) lines for brain repair in neurological disorders. However, their limitations in transferring genes to non‐dividing cells and potential oncogenic risks have rendered them relatively uncommon in preclinical studies on stroke.^15^ Lentiviral and AAV vectors are preferred for mediating stable expression. Although lentiviral vectors have twice the packaging capacity of the latter, they are more suitable for in vitro treatments, while AAV is more suitable for in vivo treatments targeting the CNS.^16^, ^17^, ^18^ Adenoviral vectors can transfer exogenous genes into the brain,^19^ but their gene expression is relatively transient and highly cytotoxic. In comparison to AAV and lentiviral vectors, which allow for sustained and stable gene expression with high safety profiles, adenoviral vectors have become relatively “outdated” in stroke research.^15^, ^20^ Therefore, in this paper, we focused on AAV vectors.

AAV is a small (~4.7 kb) single‐stranded DNA virus that was initially isolated as a pollutant in preparing simian adenovirus (Ad) in 1965.^14^ Subsequently, researchers began to conduct comprehensive and in‐depth studies on the structural basis, biological characteristics, transduction mechanisms, and immunogenicity of AAV. The wild‐type form of AAV is commonly present in human and non‐human primate (NHP) populations. As an adenovirus‐related particle, it is dependent and deficient, requiring the assistance of unrelated auxiliary viruses such as Ad or herpes simplex virus for productive replication.^15^

The AAV genome is highly simplified, featuring inverted terminal repeat sequences (ITRs) on either genome end, forming a T‐shaped structure, which is the origin of replication and packaging signals, respectively. Open reading frames (ORFs) lie between the two ITRs,^21^ where ORF rep encodes four non‐structural proteins responsible for regulating gene expression, replication, transcription, and membrane coating, while cap encodes three structural proteins that assemble to form the viral capsid.^22^ An additional ORF, nested within the cap gene, encodes the assembly activating protein, which is responsible for regulating AAV assembly.^23^ Varied processing of the ORF of the cap leads to AAV's broad and variable tissue tropism.

To facilitate gene therapy, the viral genome is replaced with the genomic therapeutic cassette of interest, which consists of the transgene and regulatory elements. This modification generates translating into a recombinant AAV (rAAV) version, utilizing helper viral genes required for replication.^22^ The rAAV vectors achieve “transduction” by receptor‐mediated endocytosis, intracellular transport, nuclear uptake, and gene expression, delivering the therapeutic gene cassette to target cells.^24^ Since rAAV is a single‐stranded genome, which is required to reach the nucleus, it must be synthesized or complemented with a complementary strand enabling gene expression, an essential step in the transduction process, which is time‐consuming and might pose limitations in terms of cell type and rAAV genome. To address these issues, researchers successfully developed self‐complementary AAV (scAAV) vectors, which possess pre‐formed double‐stranded structures that can directly enter the transcription and translation processes, thereby enhancing transduction efficiency.^25^

The use of rAAVs to target neurons in the brain was first reported by Kaplitt et al. and subsequently, rAAVs became increasingly prevalent as gene transfer vectors in the nervous system.^26^ The deletion of the rep and cap genes from the genome has reduced the risk of pathogenesis during rAAV replication. AAV vectors have the ability to transduce a wide range of terminally differentiated or non‐dividing cells, such as neurons, and can also selectively transduce astrocytes, oligodendrocytes, endothelial cells, photoreceptor cells in the retina, and dorsal root ganglia.^27^, ^28^, ^29^, ^30^ AAV is well suited for transgene delivery due to its ability to transpose both dividing and nondividing cells for long‐term stable expression with minimal risk to the subject.

The current AAV vector strategies, when combined with various tools, can achieve specific expression of various cell types in neuroscience, or whole‐body expression in animals. These tools include targeting markers like fluorescent proteins (e.g., GFP and mCherry), chemo‐genetic tools, programmable genome editing tools (e.g., mega‐nucleases and CRISPR‐Cas9), and various transgenic animals.^31^, ^32^, ^33^ This combination allows for precise delivery of transgenic effects while facilitating the mapping of neural anatomical and functional pathways, with the added advantages of spatial and temporal specificity. Moreover, with the greatly expanded ability of AAV vectors to deliver transgene libraries, different therapeutic boxes can also be carried for post‐stroke treatment, including anti‐inflammatory, anti‐apoptotic, pro‐angiogenic, axon reconstruction, neuro‐regenerative genes, and others for post‐ischemia molecular regulation. These merits advocate the AAV as a potential delivery vector for stroke gene therapy and have been extensively evaluated in many preclinical stroke models.^34^

## 3 THERAPEUTIC POTENTIAL OF AAV VECTORS IN PRECLINICAL STROKE MODELS

**FIGURE 1:**
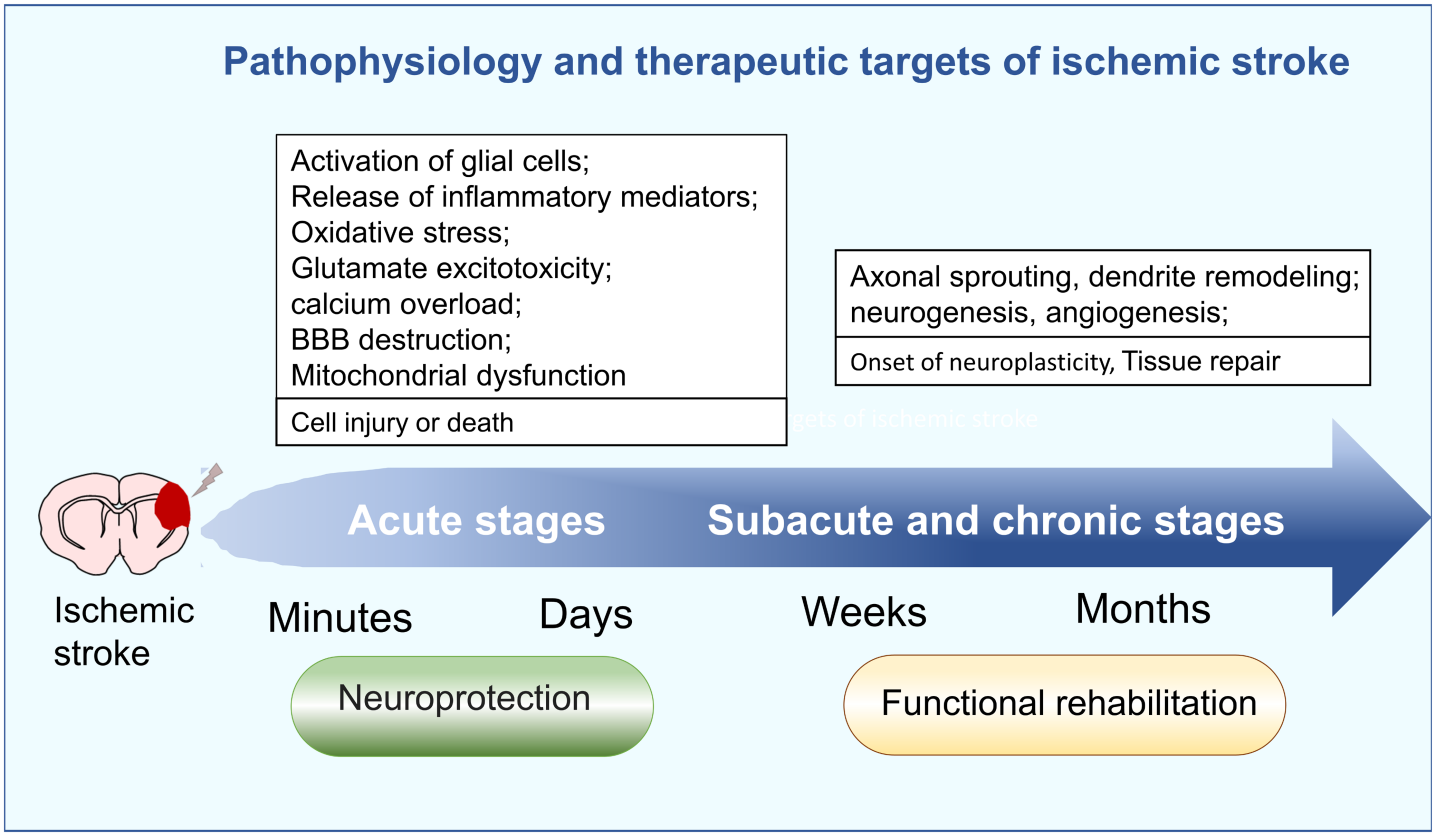
Pathophysiology and therapeutic targets of ischemic stroke.

It is pertinent to mention that different stages following a stroke have distinct pathophysiological characteristics requiring specific treatment principles (Figure 1). During the acute phase (spanning from minutes to days), activation of glial cells, the release of inflammatory mediators, oxidative stress, glutamate excitotoxicity, calcium overload, and breakdown of the blood–brain barrier (BBB) result in activating series of detrimental signaling cascades.^35^, ^36^, ^37^, ^38^ Neuroprotection is critical during this phase.^39^, ^40^ During the subacute (spanning from days to weeks) and chronic stages (spanning from weeks to months), endogenous activities such as axonal sprouting, cortical excitability changes, and neuroplasticity are enhanced. Therefore, promoting neurogenesis, excitability, tissue repair, and functional rehabilitation that facilitate behavioral recovery are the fundamental treatment principles.^41^, ^42^, ^43^

**FIGURE 2:**
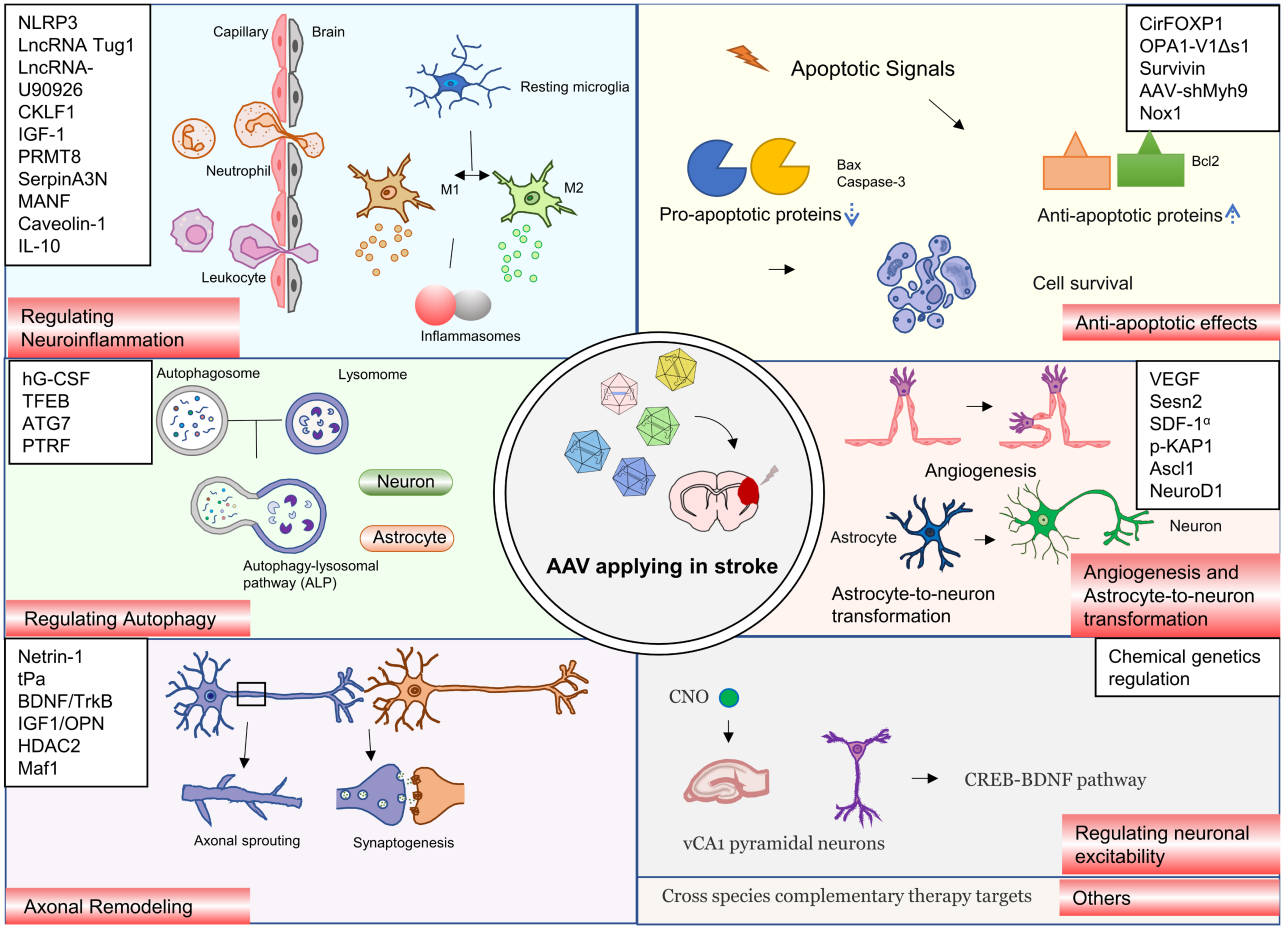
Adeno‐associated virus (AAV) vectors applying in preclinical stroke models.

The cohort of endogenous genes upregulated or downregulated by ischemic events in stroke represents a valuable resource of interest. By utilizing AAV‐mediated delivery or in conjunction with other genetic tools, overexpressing “protective” factors or knocking down “harmful” genes at a specific time that aligns with the post‐stroke therapeutic window may potentially aid in the restoration of neural function. Additionally, modulation of neuronal excitability through chemo‐genetic means has emerged as a novel means of manipulation. This section will explore the in vivo preclinical studies of AAV‐mediated ischemic stroke models. The utilization of AAV vectors in preclinical stroke models has been summarized in Figure 2, with a detailed description included in Table 1.

**TABLE 1:** Therapeutic potential of AAV vectors in preclinical stroke models.

Function	Species	Stroke model	Vector(s)	OE/KD	Time of administration (pre‐/post‐injury)	Route(s) of administration	Reference
Regulating neuroinflammatory response	Rats	tMCAO	AAV5‐GFAP‐hIGF‐1	OE	Pre‐6–8 weeks	Intracranial: cortex, striatum	[44]
	Mice	tMCAO	AAV‐shRNA‐NLRP3	KD	Pre‐2 weeks	Intracranial: cortex	[45]
	Mice	PT	AAV‐shTug1	KD	Pre‐3 weeks	Intracerebroventricular	[46]
	Rats	tMCAO	pAAV‐CMV‐Nlrp3‐3FLAG	KD	Post‐2 weeks	Intracranial: hippocampus	[47]
	Mice	tMCAO	AAV‐F4/80‐shRNA‐U90926	KD	Pre‐3 weeks	Intracranial: cortex	[48]
	Rats	tMCAO	AAV‐CKLF1	KD	Pre‐3 weeks	Intracranial: cortex	[49]
	Transgenic mice	tMCAO	AAV2/6‐CMV‐sh. SENP6	KD	Pre‐4 weeks	Intracranial: hippocampal CA1, cortex, and striatum	[50]
	Mice	tMCAO	AAV‐PRMT8	OE	Pre‐4 weeks	Intracerebroventricular	[51]
	Mice	tMCAO	AAV‐serpinA3N‐ZsGreen	OE	Pre‐4 weeks	Intracranial: striatum	[52]
	Rats	tMCAO	DJ‐1 AAV/ scramble AAV	OE	Pre‐1 month	Intracranial: cortex	[53]
	Mice	tMCAO	AAV2/5‐CAG‐Nln	OE	Pre‐2 weeks	Intracranial: striatum	[54]
	Rats	dMCAo	AAV7‐MANF	OE	Post‐1 day	Intracranial: cortex	[55]
	Rats	dMCAo	AAV1‐MANF	OE	Post‐1 day	Intracranial: cortex	[56]
	Mice	tMCAO	AAV‐Tie1‐MCS‐EGFP‐3Flag‐SV40 PolyA	OE	Pre‐3 weeks	Intracerebroventricular	[57]
	Rats	tMCAO	AAV‐IL‐10	OE	Post‐0, 3 h	Intravenous	[58]
Anti‐apoptotic effects	Mice	tMCAO	AAV‐CMV bGlobin‐EGFP‐circFOXP1 O	OE	Pre‐4 weeks	Intracerebroventricular	[59]
	Rats	tMCAO	AAV‐OPA1‐v1ΔS1	OE	Pre‐3 weeks	Intracranial: cortex, striatum	[60]
	Rats	tMCAO	AAV‐His‐survivin	OE	Pre‐3 weeks	Intracranial: striatum	[61]
	Mice	dMCAo	rAAV‐shMyh9‐GFP	KD	Pre‐4 weeks	Intravenous	[62]
	Rats	tMCAO	Nox1 shRNA/AAV	KD	Pre‐4 weeks	Intracranial: cortex, striatum	[63]
Regulating autophagy	Mice	BCAO	scAAV2‐CMV‐hG‐CSF	OE	Post‐30–60 min	Intraocular	[64]
	Rats	pMCAO	AAV2/9‐TFEB	OE	Pre‐4 weeks	Intracranial: cortex	[65]
	Mice	tMCAO	AAV‐GFAP‐ATG7	OE	Pre‐3 weeks	Intracranial: cortex	[66]
	Mice	tMCAO	AAV‐hSyn‐sh‐PTRF	KD	Pre‐2 months	Intracranial: cortex	[67]
Angiogenesis and astrocyte‐to‐neuron transformation	Rats	PT	AAV‐VEGF	OE	Post‐1 day	Microneedle patch implantation	[68]
	Rats	pMCAO	AAV‐Sesn2	OE	Pre‐2 weeks	Intracerebroventricular	[69]
	Rats	tMCAO	AAV2/9‐KAP1‐GFP	OE	Pre‐2 weeks	Intracerebroventricular	[70]
	Mice	pMCAO	AAV‐SDF‐1α	OE	Post‐1 week	Intracranial: cortex	[71]
	Mice/rats	Focal ischemic stroke	pAAV‐FLEX‐NeuroD1	OE	Post‐10 days/23–24 days	Intracranial: cortex	[72]
	Rhesus macaques	Focal ischemic stroke	pAAV‐Flex‐CAG‐ND1‐P2A‐ mCherry	OE	Post‐10, 21, 30 days	Intracranial: cortex	[73]
Axonal remodeling	Rats	pMCAO	AAV‐NT‐1	OE	Post‐1 h	Intracranial: cortex, striatum	[74]
	Transgenic mice	pMCAO	AAV5‐Tpa	OE	Post‐1 day	Intramuscular	[75]
	Rats	pMCAO	AAV‐retro‐BDNF､AAV‐retro‐TrkB	OE	Post‐1 day	Intramuscular, Intranasal	[76]
	Mice	PT	AAV2/1‐IGF1､AAV2/1‐OPN	OE	Post‐1 day	Intracranial: cortex	[77]
	Transgenic mice	PT	AAV‐retro‐Flex‐hM3Dq‐mCherry	KD	Pre‐3 days	Intracranial: cortex	[78]
	Mice	PT	AAV‐PHP.Eb shMaf1 AAV	KD	Pre‐3 weeks	Intracranial: cortex	[79]
	Transgenic mice	PT	AAV‐retro‐Flex‐GFP	–	Pre‐2 weeks	Intraspinal	[31]
Chemical genetics regulation of neuronal excitability	Mice	tMCAO	pAAV9‐CaMKIIα‐hM4D (Gi)‐mCherry/pAAV9‐CaMKIIα‐mCherry‐P2A‐Creb1	–/OE	Pre‐3 weeks	Intracranial: vCA1	[80]
Others	Transgenic mice	dpMCAO	pAAV9‐CMV‐RABEP2	OE	Pre‐3 or 10 weeks	Intracranial: cortex	[81]
	Rats	pMCAO	BDNF‐HA2TAT/AAV	OE	Post‐22–32 days	Intranasal	[82]

Abbreviations: KD, knock‐down; OE, overexpression.

### 3.1 Regulating neuroinflammatory response

The neuroinflammatory process of ischemic stroke begins within minutes of onset, involving the recruitment of peripheral immune cells, activation and phenotypic transformation of glial cells, and release of cytokines and chemokines, lasting for a prolonged time.^83^, ^84^ Microglia‐mediated inflammasome activation also contributes to the stroke‐induced inflammatory response, wherein the inhibition of inflammasomes like NLRP3 plays an important role in neuroprotection.^85^, ^86^, ^87^ In a study investigating the effects of curcumin on white matter damage after stroke, the use of AAV‐mediated NLRP3 knockdown was found to reduce pyroptosis‐related protein levels in the peri‐infarct area of mice 21 days after ischemia–reperfusion, alleviating the post‐stroke white matter lesions.^45^ Moreover, a separate study examining the effects of *Morinda officinalis* oligosaccharides on post‐stroke depression (PSD) in rats revealed that administering pAAV2/8‐CMV‐Nlrp3‐3FLAG into the hippocampus resulted in ameliorated depressive‐like behavior in PSD rats following transient middle cerebral artery occlusion (tMCAO) surgery and mitigated the hippocampal inflammation.^47^

Non‐coding RNAs (ncRNAs), including long ncRNAs taurine, upregulated gene 1 (lncRNA Tug1) and lncRNA‐U90926, as well as cytokines, have been found to play a role in brain inflammation mediated by activated microglia and neutrophils, which could result in a reduction in cerebral infarction volume and improvement of neurological function. Intraventricular infusion of AAV‐mediated Tug1 knockdown rescued damaged cortical neurons in photo‐thrombosis (PT) stroke mice 24 h after surgery, reducing the microglial accumulation.^46^ In a tMCAO mouse model, silencing of microglia U90926 resulted in a decreased influx of neutrophils at 24 h post‐ischemia.^48^ Furthermore, downregulating the expression of chemokine‐like factor 1 (CKLF1) in the ischemic brain area through shRNA‐mediated knockdown via AAV vectors resulted in reduced migration and infiltration of neutrophils.^49^

The upregulation of post‐ischemic neuroprotective factors through AAV‐mediated gene therapy can promote functional recovery after stroke. It has been reported that insulin‐like growth factor (IGF)‐1, a nutritional component, can be synthesized and released in astrocytes, promoting neuronal survival, neuroplasticity, and inhibiting CNS degeneration.^88^ Altered microenvironmental signaling that shifts the phenotype of microglia from pro‐inflammatory M1 to anti‐inflammatory M2 can exert neuroprotective effects.^89^ Injecting of AAV5‐GFAP‐hIGF‐1 into the cortex and striatum of middle‐aged rats 6–8 weeks prior to MCAO surgery facilitated the expression of hIGF‐1 in glial fibrillary acidic protein (GFAP) and was found to promote activation of regulatory T cells and to infiltrate M2 macrophages in the ischemic hemisphere, leading to improved neural function within 24 h after stroke.^44^ Similarly, protein arginine methyltransferase 8 (PRMT8) is downregulated in ischemic stroke models, thus continuous AAV‐PRMT8 injection into the lateral ventricle of tMCAO mice for 7 days not only upregulated Lin28a but also promoted M2 polarization of microglia and neuronal survival, and improved histopathological changes in the brain.^51^ The overexpression of serine proteinase inhibitor A3N (serpinA3N) significantly reduced the size of cerebral infarcts in tMCAO mice 24 h after surgery and indirectly inhibited neuroinflammation.^52^ In addition, overexpression of DJ‐1 or Nln through AAV gene therapy can also reduce stroke volume to varying degrees, inhibit pro‐inflammatory cytokines, promote the expression of anti‐inflammatory factors, and alleviate stroke injury.^53^, ^54^ For this purpose, Mätlik K et al. injected AAV7‐mediated mesencephalic astrocyte‐derived neurotrophic factor (AAV7‐MANF) into the peri‐infarcted area of rats 2 days after distal middle cerebral artery occlusion surgery, which resulted in an increased number of phagocytic immune cells in the subcortical area 4 days post‐surgery with enhanced behavioral recovery.^55^ Subsequently, they described the molecular spectrum of the ischemic stroke peri‐infarct area in another study, establishing a resource for further stroke research.^56^

The concept of cerebral thrombo‐inflammation involves the interaction between thrombosis and inflammation, which may synergistically drive the progression of ischemic stroke, with endothelial dysfunction being an early prerequisite.^90^, ^91^ Caveolin‐1 is a membrane lipid raft scaffold protein that plays a crucial role in neuronal survival.^92^ At tMCAO‐24 h, the levels of endothelial caveolin‐1 (Cav‐1) in the endothelium and serum of mice were found to decrease. Specific enhancement of Cav‐1 expression by AAV‐Tie1‐Cav‐1 significantly reduced the infarct volume in both wild‐type and Cav‐1^−/−^ tMCAO mice. This intervention resulted in a concordant attenuation of microvascular deterioration and alleviation of thrombo‐inflammation.^57^

The AAV‐mediated gene modification also plays a role in the anti‐inflammatory process of transplanted mesenchymal stem cells (MSCs). The IL‐10 gene modification of MSCs in the acute‐phase transplantation reduced the levels of pro‐inflammatory cytokines after tMCAO in Sprague–Dawley (SD) rats, enhanced the neuroprotective effect of MSCs, and prolonged the therapeutic time window of MSC transplantation.^58^ It is pertinent to mention that AAV‐mediated transplantation of MSC/IL‐10 resulted in the upregulation of IL‐10 levels in the ischemic hemisphere while not affecting serum IL‐10 levels, potentially reducing the risk of systemic adverse reactions.

### 3.2 Anti‐apoptotic effects

The ischemia induces various morphological changes in the mitochondria, resulting in oxidative stress buildup facilitating apoptotic pathway.^93^ Acute ischemic stroke drives the pathological progression via circular RNAs. Studies have demonstrated that AAV‐mediated circFOXP1 overexpression regulates STAT3/apoptotic signaling, significantly reducing the apoptotic signal in the infarct area of tMCAO mice, resulting in enhanced expression of the anti‐apoptotic protein (Bcl2) and inhibition of pro‐apoptotic proteins (Bax and caspase‐3), thereby improving functional recovery.^59^ Optic atrophy 1 (OPA1) is a mitochondrial regulatory gene with the potential to alleviate neuronal cell apoptosis and mitochondrial dysfunction. Overexpression of OPA1‐v1ΔS1 in adult male SD rats can resist neuronal apoptosis and oxidative stress and alleviate mitochondrial dysfunction.^60^ The overexpression of the survival of protein survivin in the ischemic border zone may also improve ischemic injury in rats through an anti‐apoptotic mechanism.^61^

Non‐muscle myosin heavy chain IIA (NMMHC IIA) is a member of the cellular skeleton that induces neuronal apoptosis. NADPH oxidase (Nox1) protein can generate a large amount of reactive oxygen species, inciting tissue damage. It has been observed that injecting AAV‐shMyh9 via the tail vein downregulated NMMHC IIA expression and inhibited I/R‐induced NMMHC IIA‐actin interaction and the caspase‐3/ROCK1/MLC signaling pathway.^62^ Similarly, stereotactic injection of AAV2‐mediated Nox1 enhancement in the peri‐infarct area promoted the survival of new neurons and improved neurological dysfunction.^63^ Thus, it is evident that both of these agents exerted their effects via anti‐apoptotic mechanisms.

### 3.3 Regulating autophagy

Autophagy in ischemic stroke is regarded as a double‐edged sword, where autophagy regulation could help reduce ischemic injury.^94^ Human granulocyte colony‐stimulating factor (hG‐CSF) is a neurotrophic factor having neuroprotective effects. However, its short biological half‐life of 4 h and repeated use might precipitate adverse anti‐drug antibody reactions. Therefore, long‐acting delivery carriers are pivotal for sustaining the payload release and reducing dosing frequency. A study reported the transfection of scAAV2 as a vector carrying the hG‐CSF gene (a growth factor) into the brains of mice with global ischemia, which resulted in the downregulation of the expression levels of autophagy proteins (Beclin 1, p62, and LC3‐ll) in the damaged brain and improved overall behavioral functioning.^64^ Similarly, transcription factor EB (TFEB) is another major regulatory factor of the autophagy–lysosomal pathway (ALP); hence, targeting neurons with TFEB can rescue ALP dysfunction and alleviate ischemic injury in rats after permanent middle cerebral artery occlusion (pMCAO).^65^ Additionally, the autophagy‐related gene (ATG7) is involved in autophagy induction and the formation of autophagosomes. AAV2/9‐GFAP‐ATG7 was found to upregulate autophagy in astrocytes of C57/BL6 mice after ischemia/reperfusion for 24 h, exerting a neuroprotective effect.^66^ The polymerase I and transcript release factor (PTRF) is a cytoplasmic protein involved in the formation and function of caveolae. An AAV vector driven by human synapsin (hSyn) promoter was found to downregulate PTRF in ischemic penumbra region neurons, regulating the PTRF/PLA2G4A axis to reduce autophagy in neurons and alleviate brain damage in tMCAO mice.^67^

### 3.4 Angiogenesis and astrocyte‐to‐neuron transformation

Inducing angiogenesis can promote functional outcomes in stroke patients. Within 24 h of an ischemic attack in adult rats, local delivery of AAV‐VEGF into the ischemic core and penumbra using micro‐needles ensured continuous release, achieving uniform and efficient transduction, where enhanced functional angiogenesis and neurogenesis was observed due to VEGF overexpression after 8 weeks of treatment.^68^ Overexpression of stress response protein Sesn2 in photochemical cerebral ischemia model (PM) rats also upregulated VEGF levels and enhanced downstream factors involved in angiogenesis.^69^ The AAV‐mediated transfer of the stromal cell‐derived factor‐1α (SDF‐1α) gene to the area surrounding the infarct in pMCAO mice significantly reduced brain atrophy and promoted neurogenesis and angiogenesis. KAP1 is a transcriptional co‐factor.^95^ In the rat MCAO model, high expression of p‐KAP1 gene through AAV2/9 mediation was achieved by injecting it into the lateral ventricle 2 weeks prior to onset of stroke. Further studies demonstrated that p‐KAP1 could bind with proliferating cell nuclear antigen (PCNA) and inhibit the binding of E3 ubiquitin ligase CUL4A with PCNA, thereby enhancing the survival and proliferation of endogenous NSCs.^70^

The application of gene therapy to induce the transforming astrocytes to neurons in the damaged brain after ischemic stroke in adult animals has been studied as a potential strategy for promoting brain recovery. Research has demonstrated that targeted expression of achaete‐scute complex homolog‐like 1 (Ascl1) through the use of GFAP‐AAV vectors can convert astrocytes in the dorsal midbrain of mice into fully functional induced neurons in vivo.^96^ In ischemic injury models involving adult mice and rats, the utilization of the engineered AAV Cre‐FLEX system enabled the ectopic expression of the neurotranscription factor NeuroD1 in reactive astrocytes. This groundbreaking approach effectively promoted the conversion of astrocytes into neurons, leading to the restoration of neuronal function that had been compromised as a consequence of the injury.^72^ Furthermore, Ge et al. studied cell transformation in NHPs rhesus monkeys based on AAV NeuroD1 gene therapy, which is currently the only non‐rodent animal model reported. Results showed that upregulation of NeuroD1 could convert 90% of endogenous infected astrocytes into functional neurons, providing more valuable information than rodents.^73^

### 3.5 Axonal remodeling

Promoting axonal rewiring in subacute and chronic stages after stroke can impact clinical treatment outcomes,^97^ where persistent and stable expression characteristics of AAV make it suitable for promoting neural plasticity after injury. Secreted protein Netrin‐1 is an axon guidance molecule, and overexpression of AAV‐mediated Netrin‐1 in rats after pMCAO was found to increase the expression of the Netrin‐1 receptor DCC, activated the JNK1/c‐Jun pathway, and promoted synapse formation, axonal regeneration, and neurological function recovery in the subacute phase of stroke.^74^ Tissue plasminogen activator (tPA), administered intravenously to dissolve blood clots after a stroke, has been limited by its short time window. However, in pMCAO mice, AAV injected intramuscularly into the muscles of the paralyzed limbs 24 h after surgery resulted in sustained overexpression in denervated spinal motor neurons for at least 28 days. This method facilitated corticospinal tract (CST) axon remodeling in the cervical spinal cord, where behavioral outcomes were significantly correlated with neural remodeling. The tPA was not used as a fibrinolytic agent but as a neural recovery agent stimulating axon remodeling.^75^ Additionally, combinational therapy involving intramuscular AAV‐BDNF and intranasal AAV‐TrkB administered 1 day after surgery promoted motor function recovery, axon reorganization, and synaptic plasticity in the spinal cord denervated gray matter in pMCAO rats for 8 weeks.^76^ Injecting AAV into the contralateral somatosensory motor cortex in mice 3 days post‐unilateral PT stroke succeeded in co‐expressing two soluble proteins, IGF1 and osteopontin (OPN), which promoted CST sprouting in the spinal cord and subcortical regions and restored behavioral function.^77^ Histone deacetylase 2 (HDAC2) is a negative regulator of neural plasticity, and virus‐mediated HDAC2 downregulation promoted axon sprouting and enhanced the expression of neurotrophic factors (NGF and BDNF) and neural plasticity‐related proteins (PSD95 and synapsin), enhancing neural plasticity.^78^ Similarly, Maf1 is a transcriptional regulator that suppresses spontaneous neural repair, where knocking down Maf1 using AAV‐mediated delivery in a mouse PT stroke model enhanced neural plasticity and functional recovery.^79^

Combining rehabilitation training with sensory (peripheral proprioceptor) stimulation has been shown to promote motor recovery after ischemic stroke.^31^ To this end, scholars injected the Cre‐dependent AAV into the spinal cord of PV‐Cre‐Ai14 mice and used chemo‐genetic methods to activate proprioceptors chronically. The results showed that the combinational therapy promoted mTOR activity in layer five cortical neurons and axonal sprouting in the spinal cord, significantly promoting skilled movement and observing motor function recovery.^31^

### 3.6 Chemical genetics regulation of neuronal excitability

The designer receptors exclusively activated by designer drugs strategy uses AAV vectors and clozapine‐N‐oxide (CNO) to modulate neuronal excitability in a cell‐type‐specific and reversible manner inside the body. Both AAV and CNO have been tested in humans, providing promising prospects for the potential applications of chemical genetics in clinical treatments.^98^ Inhibiting glutamatergic neurons in vCA1 through chemical genetics in the early stages (4–7 days) after MCAO in mice can reduce neural defects, pain perception, anxiety, and depressive behaviors caused by cerebral ischemia. In addition, activation of the CREB‐BDNF pathway in hippocampal pyramidal neurons of cerebral ischemic mice significantly alleviated cerebral ischemia‐induced neurological dysfunction, mechanical allodynia, anxiety, and depression.^80^

### 3.7 Others

A study was conducted to develop an in vivo evaluation system to screen and validate the function of cross‐species RABEP2 mediated by AAV vectors in Rabep2 knockout (KO) mice.^81^ The results indicated that both mouse and human RABEP2 could rescue the ischemic stroke volume and collateral vessel phenotype in Rabep2 KO mice, while human RABEP2‐coding mutations significantly impaired the rescue of collateral vessel density and infarct volume phenotypes. This cross‐species complementarity expanded the range of therapeutic targets for stroke in humans.

Furthermore, Chen et al. designed a novel fusion gene HA2TAT‐BDNF and constructed BDNF‐HA2TAT/AAVBDNF‐HA2TAT/AAV in combination with AAV, which improved depressive‐like behavior in PSD model rats after nasal administration.^82^

## 4 MANIPULATION AND PROSPECTS OF AAV VECTORS IN STROKE MODELS

**FIGURE 3:**
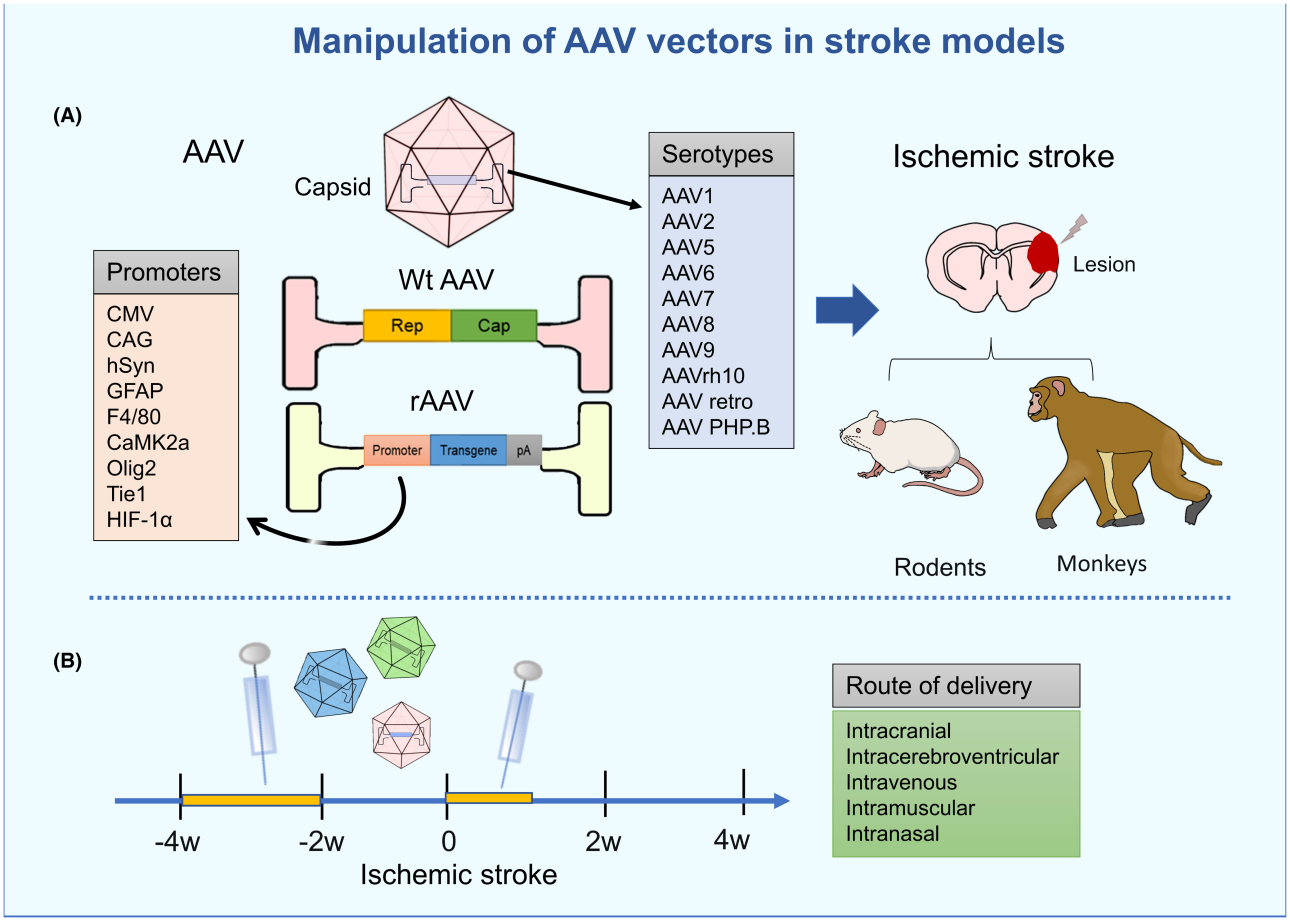
Manipulation of adeno‐associated virus (AAV) vectors in ischemic stroke models. (A) The serotypes and promoters commonly employed. (B) The delivery time and strategy commonly utilized.

AAV has played a significant role in identifying an increasing number of therapeutic targets and in translating discoveries into practical applications. When considering the utilization of AAV for stroke treatment, the question arises as to whether the target gene we desire can be processed by AAV. How can specific and efficient expression of the target gene be achieved, taking into account timing and location? Are there potential associations with adverse reactions? In fact, the enhancement of cellular or tissue tropism and the control of transgene expression can be achieved through the modification of engineering features of AAV, such as therapeutic gene boxes, capsids, and regulatory elements. The strategies for manipulating AAV vectors in preclinical studies of stroke models have been shown in Figure 3.

### 4.1 Therapeutic gene toolbox

The development of sequencing technology and bioinformatics has contributed to understanding the pathogenesis of ischemic stroke, selecting therapeutic targets, and establishing biomarkers, while AAV vectors have contributed to translating gene discoveries into therapeutic interventions. Genes involved in the progression of ischemic stroke include various neurotrophic factors (MANF, BDNF),^55^, ^56^, ^82^ transcription factors (ATG7, NeuroD1, and CREB),^66^, ^73^, ^80^ inflammasomes NLRP3,^45^, ^47^ LncRNAs (lncRNA Tug1 and lncRNA‐U90926),^46^, ^48^ cytokines (CKLF1, SDF‐1α, IL‐10, hG‐CSF, and VEGF),^49^, ^58^, ^64^, ^68^, ^71^ neuroplasticity regulator (tPA, HDAC2, and Maf1),^75^, ^78^, ^79^ circular RNAs,^59^ among others.

One inherent demerit associated with AAV is limited packaging, that is, 4.7 kb of exogenous DNA, thereby limiting cloning capacity.^16^ When exceeding the AAV vector's packaging capacity, using self‐complementary double‐stranded DNA in the vector genome may allow the delivery of larger proteins.^99^ In addition, some molecules not easily targeted by conventional drugs can be inhibited by developing targeted therapeutic genes using RNA interference and other antisense strategies.^100^ For example, in stroke models, AAV delivery of shRNA against NLRP337,^45^ Tug139,^46^ and lncRNA‐U9092641^48^ resulted in the silencing of these genes and protected against the ischemic process.

### 4.2 Serotypes

Each AAV serotype possesses a unique surface antigen, and the engineering design of the AAV capsid can produce novel AAV variants with broader organ and tissue tropism.^101^ Presently, multiple serotypes exhibit the ability to target the CNS. The AAV1, 2, 5, 6, 7, 8, 9, rh 10, retro, and PHP.B are used more in preclinical stroke research.^102^, ^103^ After injection, most transgenes are expressed around the injection site, with not only main neuron tropism but also transduction of astrocytes or microglia.^50^, ^52^, ^71^, ^79^ AAV2 is currently considered the most extensively studied serotype, but AAV9 appears to be used most frequently in ischemic stroke.^59^, ^62^, ^70^, ^73^, ^80^, ^81^ When delivered intravenously, AAV9 can traverse the BBB to target newborn neurons and adult glial cells,^29^ and drive axonal transport.^104^ A newly engineered capsid, AAV‐PHP.B, has also been used in stroke research.^105^ According to reports, after intravenous injection, it can cross the BBB more efficiently than AAV9 in mice,^106^ but its transduction efficiency in the CNS of primates was similar to that of AAV9.^107^ The application of engineered AAV provides a non‐invasive alternative strategy for gene delivery.

Most AAV vectors are transported in an anterograde, non‐synaptic manner, with relatively weaker retrograde transduction capability. However, rAAV‐retro, a variant of AAV, has strong retrograde transduction efficiency, allowing it to be transported in a retrograde, non‐synaptic manner to projecting neurons. The retrograde transduction efficiency of rAAV‐retro is two orders of magnitude higher than other common serotypes, and even comparable to the classic retrograde tracer, Fluorogold. In studying neural circuits or functions, rAAV‐retro gene delivery can be used alone or in combination with the Cre recombinase driver system. When injected into PV‐Cre mice's spinal cord, AAV‐retro or AAV6 was reported to preferentially label DRG neurons,^108^ but the retrograde targeting ability of AAV6 was much lower than that of rAAV‐retro.^31^ In addition, muscle injection of AAV‐retro can effectively transduce lower motor neurons in the spinal cord and brainstem, providing a simple and practical method for stroke gene delivery.^76^, ^109^

### 4.3 Promoters

Unlike the inherent organ and tissue tropisms of AAV vectors, the selection of specific promoters in the construct can significantly affect the transgene expression pattern.^110^, ^111^, ^112^ In stroke research, ubiquitous promoters such as the non‐specific cytomegalovirus (CMV) or chicken‐β‐actin hybrid (CAG) are expressed in most CNS cells, primarily neurons and some scattered astrocytes.^79^, ^103^, ^113^ Various promoters can drive specific transgene expressions, such as the hSyn promoter driving neuron‐specific gene expression,^53^, ^75^, ^76^, ^106^, ^114^ the GFAP promoter driving astrocyte‐specific expression,^44^, ^66^, ^73^, ^88^ the F4/80 promoter driving microglia‐specific expression,^44^, ^66^, ^73^ the CaMK2a promoter driving glutamatergic neuronal expression,^80^ and the Olig2 promoter driving oligodendrocyte‐specific expression.^115^ Additionally, regulatory elements such as the Tie1 promoter can drive brain endothelial cell‐specific expression^57^, ^116^ and the HIF‐1α promoter is regulated under hypoxic conditions.^49^, ^117^

Specific promoters combined with certain serotypes may result in different characteristics, such as selectively transducing neurons or astrocytes^118^ or enhancing tissue‐specific promoter strength while maintaining promoter specificity.^103^ Some researchers have found that in vivo gene expression was not different between AAV2 combined with the CBA or CBh promoters in cases where the vector components were identical. In contrast, different in vivo gene expression was observed between AAV9 and these two similar promoters.^119^ The AAV vectors have broad tropism, different serotypes, and promoter combinations, contributing to their efficient and specific sites and cell targeting. However, this may also complicate the gene expression profile, leading to unknown and novel characteristics in ischemic stroke.

### 4.4 Delivery strategies

Due to its persistent and stable expression, AAV vectors carrying genes are often delivered in a single dose. This resolves the clinical use limitation of certain drugs like human G‐CSF (hG‐CSF), which requires repeated administrations due to its short plasma half‐life.^64^ AAV gene expression typically manifests within 2–3 weeks after delivery. In stroke research, AAV delivery before ischemia is often used, while AAV delivery after stroke onset is more suitable for subacute and chronic phase treatment.

To circumvent the BBB, researchers often precisely select stereotactic coordinates based on the anatomical characteristics of animals and perform local directional injection of viral vectors for delivery. Various administration strategies, such as viral titer, injection dosage, injection depth, and individual differences, may affect the transduction efficiency of AAV vectors. In preclinical stroke studies, administration during the 2–4 weeks before ischemic surgery (some studies chose 6–8 weeks before stroke^44^, ^49^), including stereotactic injection and intravenous injection of the cortex, ventricles, hippocampus, and striatum, is often used for acute phase treatment. Transgene expression after brain parenchymal injection generally occurs around the injection site, with less expression farther away. This approach enables site‐specific expression of AAV vector genes with relatively low dosages. In addition, injecting AAV vectors into the cerebrospinal fluid (CSF) through ventricular injection can achieve widespread expression in the brain. While cisternal or intrathecal injection is also possible, there is currently limited research of this nature in the context of ischemic stroke. Both cisternal and ventricular injection procedures are invasive. When intrathecally injected, the delivery dose is generally lower than that of intravenous injection and is related to clinical applications.^120^ After injection, transgenes can be transduced into neurons and glial cells in the brain and spinal cord, but the highest transduction efficiency is near the injection site,^121^ so this may not be the optimal choice for ischemic stroke. In addition, administering before the onset of cerebral ischemia is unpredictable, and both brain parenchymal and CSF injection procedures are invasive, with poor diffusion and potential for introducing infection, making them difficult to use in clinical settings. However, they are essential in preclinical research of cerebral ischemia treatment mechanisms.

Non‐invasive vector delivery methods such as intravenous injection or intramuscular injection are preferred for clinical applications. AAV9 can cross the BBB and be delivered through tail^58^, ^62^ or neck vein injection^113^ in stroke models. In preclinical research, ocular and nasal administration routes have also been mentioned. In the transient bilateral carotid artery occlusion mouse model, a single dose of scAAV2‐CMV‐hG‐CSF was applied to the left eye, resulting in successful detection of upregulated exogenous hG‐CSF in the brain 4 days after transfection.^64^ Similarly, after nasal administration, exogenous BDNF or TrkB can be delivered to the CNS through the nasal–brain pathway for expression.^76^, ^82^ These strategies can be administered within 0–3 h after brain ischemia occurs,^54^, ^58^, ^74^ such as intravenous injection of IL‐10 gene‐transferred MSCs at 0 or 3 h after reperfusion in rats, which extended the treatment time window of MSC transplantation from 0 to 3 h.^58^ Delayed administration, ranging from 1 day to 1 month after ischemia, is more commonly used to promote functional recovery in the subacute and chronic stroke phase,^75^, ^76^, ^77^ thus laying the foundation for clinical translation. In summary, for different types and stages of stroke, individualized selection of injection location and dosage should be made based on the condition and treatment goals to maximize the therapeutic effect and minimize potential adverse effects. This section has also been presented in Table 1.

### 4.5 Other conditional expression strategies

Other conditional expression strategies can be achieved by combining AAV strategies with genetic tools, such as knock‐in driver mouse lines, to achieve gene specificity and reliable expression. Injecting the Cre‐dependent AAV‐retro virus into the spinal cord of PV‐Cre‐Ai14 mice with photothrombotic stroke targets about 74% of PV sensory neurons.^31^ Researchers have also found that by infecting the mouse brain with rAAV, these recombinases can be delivered in a GFP‐dependent manner to label specific cell populations.^122^ The recombinase CreER or FlpER induced by tamoxifen and the tetracycline (Tet) induction system packaged into rAAV can achieve transient expression at specific time points.^123^ Although currently limited to rodent studies, these strategies hold excellent great potential in post‐stroke biological research.

### 4.6 Host response

Ideally, the delivered therapeutic gene should integrate accurately into the target cells and generate the lowest possible undesired immune reactions in the population. Currently, reports have emerged on severe adverse events related to treatment. Unexpected toxicities, such as sensory neuron degeneration, liver damage, coagulation dysfunction, shock, and even death, have been observed in different species, including NHPs and piglets, following the administration of AAVhu68 (a variant of AAV9) at a dose similar to that used in the clinical trial for spinal muscular atrophy.^124^ In addition, deaths have also occurred in patients with myotonic dystrophy.^125^ Compared with rare diseases with no available treatments, the safety of AAV in stroke treatment needs to be evaluated. It is known that most people have been found to have anti‐AAV‐neutralizing antibodies,^126^ which may reduce the transduction efficiency of AAV vectors in the body. Increased usage of vectors may also result in cellular toxicity and integration of viral DNA into the host genome. Although studies have shown that transferring AAV vectors to MSCs and then performing intravenous injections in a rat model of stroke can avoid the risk of AAV‐DNA integration,^58^ while another study demonstrated that implanting microneedles loaded with AAV‐VEGF into the rat brain cortex does not exacerbate ischemia‐induced brain inflammation.^68^ However, research on the immune responses in the brain and the peripheral organ transduction effects following virus injection into a stroke model is still limited, despite being an important consideration before clinical application.

### 4.7 Challenges and prospects

As mentioned earlier, the transduction efficiency of AAV‐PHP.B in the CNS of rodents and NHPs is not consistent. Several adverse reactions noted during human clinical trials were not present in preclinical studies. These findings indicate numerous distinctive features between rodents and primates, highlighting the importance of selecting appropriate preclinical models for effective evaluation of therapeutic efficiency. This challenge may arise in all CNS studies involving AAV vectors. In preclinical studies, the transgenic expression pattern, levels, and immune‐inflammatory response following viral vector administration remain largely unknown. Currently, most preclinical studies of AAV gene therapy for stroke involve rodents and direct stereotactic injection into brain parenchyma or CSF, with most injections performed prior to the onset of ischemic injury, which poses a challenge to the future determination of the feasibility of these strategies in humans.

In addition to intravenous administration, future research may support simple delivery strategies such as intranasal or intramuscular administration of viral vectors, with the potential use of biomaterials.^88^ AAV, combined with Cre‐dependent fluorescent reporters and other methods, is a mostly non‐invasive or minimally invasive therapy that can be used for high‐intensity labeling and observation in specific anatomical locations. This can also overcome the characteristic of AAV being difficult to deliver across synapses, whether in the anterograde or retrograde direction. In addition, this strategy combined with whole‐brain imaging and reconstruction equipment such as fluorescence micro‐optical sectioning tomography may be able to reconstruct the projection of individual neurons or the entire neural circuitry of the brain.^127^, ^128^ AAV, combined with chemical genetics technology, also provides a way to regulate neuronal activity.

In neuroscience research, the biological characteristics of various AAVs have been extensively studied, and the FDA has approved Luxturna for treating RPE65‐related Leber congenital amaurosis and Zolgensma for treating spinal muscular atrophy.^129^, ^130^ Although there are relatively few studies on the application of AAV in ischemic stroke, and quantitative evaluations of transduction efficiency and observation of adverse reactions in disease states are limited, many research achievements in the relatively mature field of CNS research can provide reference and inspiration for gene therapy research in ischemic stroke. For instance, Khodanovich MY et al. utilized the doublecortin promoter (pDCX) in a stroke model of adult male SD rats, overexpressing tissue‐specific ferritin using AAV vectors. Through non‐invasive visualization of neurogenesis using magnetic resonance imaging, this study provides a novel imaging modality for use as a diagnostic tool in stroke therapy research.^131^

While pursuing efficiency, a balance with safety must also be maintained, with more preclinical research and safety assessment required to ensure the reliability and safety of clinical treatment. In summary, the combination of novel engineered capsids with safe and specific injection methods, along with cross‐study of AAV applications in ischemic stroke and other CNS diseases, holds the promise of achieving efficient and specific AAV delivery to patients with corresponding indications for ischemic stroke.

## 5 CONCLUSION

In summary, the various insights into the molecular mechanisms underlying ischemic stroke have shown promising results in preclinical studies of AAV‐mediated gene therapy in animal models of ischemic stroke. Different principles of AAV therapy apply at different time points after stroke onset. Given the flexible diversity of viral vectors, no single vector can be applied to all applications, necessitating further exploration to identify suitable viral vectors for specific indications. By using parental viruses to develop various new techniques, AAV can be designed more flexibly for accurate service to stroke patients in terms of improved engineering characteristics, specifically targeted vectors, optimized production processes and delivery strategies, and reduced adverse reactions, thereby rendering it becoming a versatile tool for therapeutic applications and biological research. Carrier‐mediated viral vector delivery may cause adverse events, as observed with other targeted drugs. Fortunately, over the past few years, a series of activities have been carried out in AAV vector design and application research, striving to reduce the potential risks associated with using AAV vectors.

The many challenges faced in preclinical research highlight the need for deep exploration. Differences in anatomical characteristics, immunogenicity, disease pathogenesis, and other factors exist between primate and rodent models, requiring the selection of more appropriate preclinical models for efficacy and safety evaluations. Many challenges make the clinical application of AAV gene therapy in ischemic stroke difficult, requiring careful and prolonged verification. Nevertheless, in the grand scheme of things, it still holds promising prospects as an exceptional tool for biological applications and research.

## AUTHOR CONTRIBUTIONS

Jing Wang wrote the manuscript. Leilei Mao and Baoliang Sun revised the manuscript with input from all authors. Jingyi Sun, Mengna Zhu, Lina Feng, and Mingfeng Yang helped to write the manuscript.

## CONFLICT OF INTEREST STATEMENT

The authors declare no conflicts of interest.

## Data Availability

Data sharing is not applicable to this article as no new data were created or analyzed in this study.
